# Crotofolanes, rearranged crotofolanes, and a novel diterpene: isocrotofolane from *Croton cascarilloides*, collected in Okinawa

**DOI:** 10.1007/s11418-023-01698-7

**Published:** 2023-04-21

**Authors:** Susumu Kawakami, Hideaki Otsuka

**Affiliations:** Department of Natural Products Chemistry, Faculty of Pharmacy, Yasuda Women’s University, 6-13-1 Yasuhigashi, Asaminami-ku, Hiroshima, 731-0153 Japan

**Keywords:** *Croton cascarilloides*, Euphorbiaceae, Crotofolane, Rearranged crotofolane, Isocrotofolane, Biological activity

## Abstract

To this day, since about 50% of all medicines are derived from natural sources, natural product chemistry, especially the search for biologically active natural components, remains extremely important (Newman and Cragg in J Nat Prod 83:770–803, 2020). In this review, we deal with our continuing research work for promising constituents from plants collected in the Ryukyu Archipelago. The isolation of islands in the archipelago by the sea or by straits gives rise to endemic plant species that are unique to the islands. The structural diversity of the constituents produced by this unique flora is of great scientific interest in various aspects, including chemical structures, biosynthesis, and biological activities. The components from this structural diversity have great potential as new pharmaceutical seeds. In our continuing studies, we have successfully investigated new but extremely unusual diterpenoids: crotofolanes and their rearranged varieties (nor-crotofolane, trinor-crotofolane, neocrotofolane) and a glycoside with a new skeletal diterpenoid (isocrotofolane glucoside) from *Croton cascarilloides*. This review summarizes our reports on the investigation of crotofolanes as well as those on crotofolanes by other research groups.

**Graphical Abstract:**

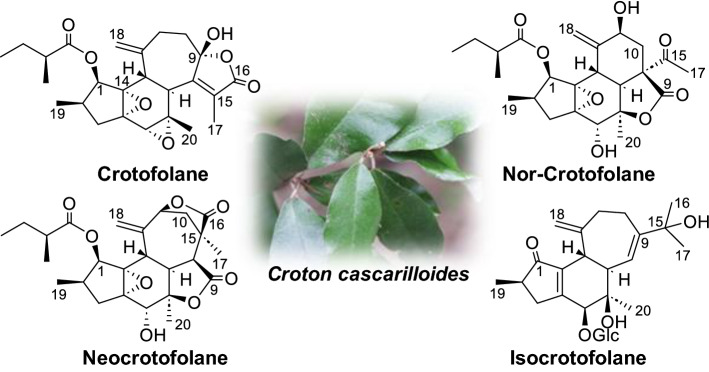

## Introduction

The Ryukyu Archipelago, which extends about 1000 km from the main island of Kyushu to the Sakishima (Yaeyama) Islands, is crossed by the Watase Line south of Yakushima Island and the Hachisuka’s Line south of Okinawa Main Island, dividing their fauna into unique areas. Similarly, their flora is divided into the Sohayaki (Kyushu) and Ryukyu regions at the Watase’s Line. There grow also many subtropical and tropical plants native to Ryukyo region. The Sakishima Islands, several hundred kilometers away from the south end of Okinawa Main Island, have a tropical-like flora, including mangroves, and the unique floral resources of the Miyako, Ishigaki, and Iriomote Islands are highly valuable as research materials for natural products chemistry. The components of structural diversity produced from plant resources in the Ryukyu Archipelago are far beyond the imagination of mankind, and the isolation of novel components is expected. Based on this point of view, the authors have been conducting research on natural products chemistry with novel molecular structures and remarkable biological activities from plants native to the Ryukyu Archipelago.

In this paper, we reviewed the chemical structures of unusual new diterpenes obtained in the course of our research: crotofolane and its rearranged varieties (nor-crotofolane, trinor-crotofolane, neocrotofolane) and a new skeletal diterpene glycoside (isocrotofolane glucoside). A summary of reports on crotofolane by other research groups is given as well.

## Crotofolane

We have studied the diversity of subtropical and tropical plant resources that produce unexpected constituents. In particular, we have searched for unutilized plant resources in the Ryukyu Archipelago for new components, and conducted bioassays to search for the biologically active components contained in them. In the course of this research, we succeeded in isolating some diterpenoids with a skeletal structure “crotofolanes,” which have rarely been reported, from *Croton cascarilloides*, collected in June 2004 at Kunigami-gun, Okinawa, Japan.

**Fig. 1:**
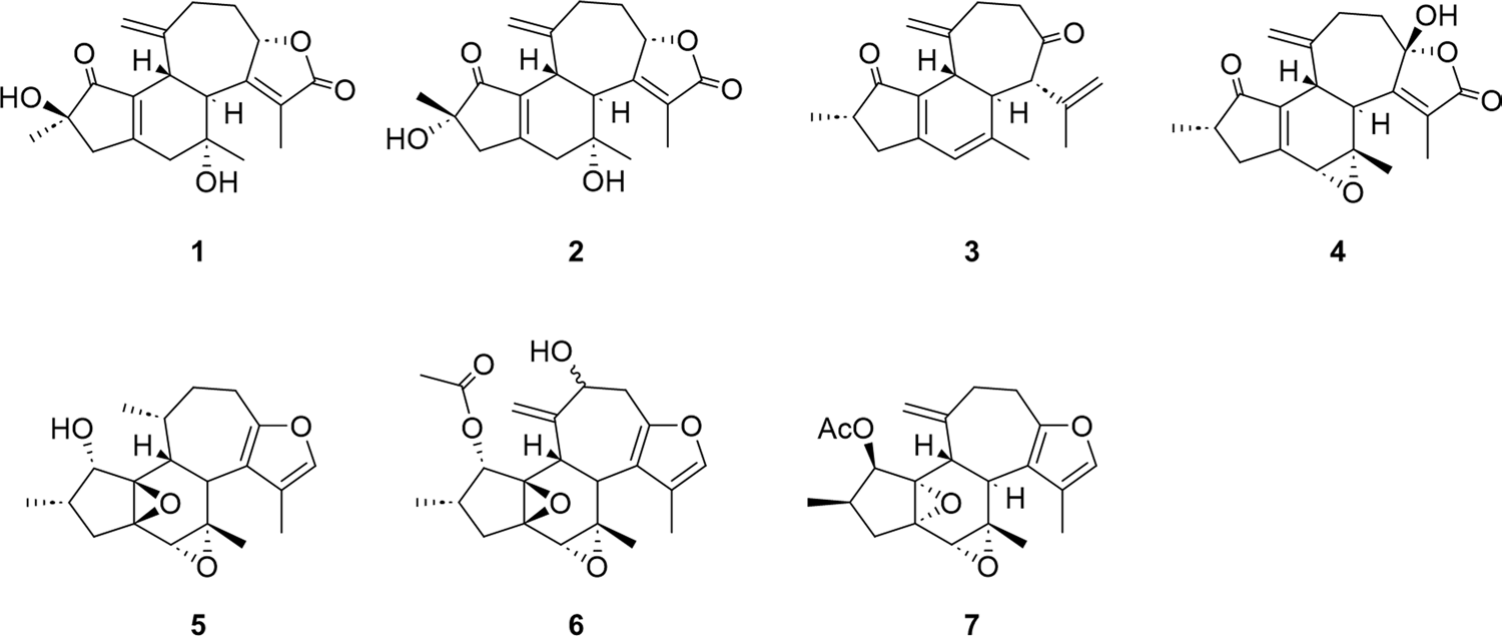
Previously reported structures of crotofolanes: crotofolin A (**1**); crotofolins B, C, and E (**2**‒**4**); crotoxides A and B (**5**, **6**); and crotohaumanoxide (**7**)

**Fig. 2:**
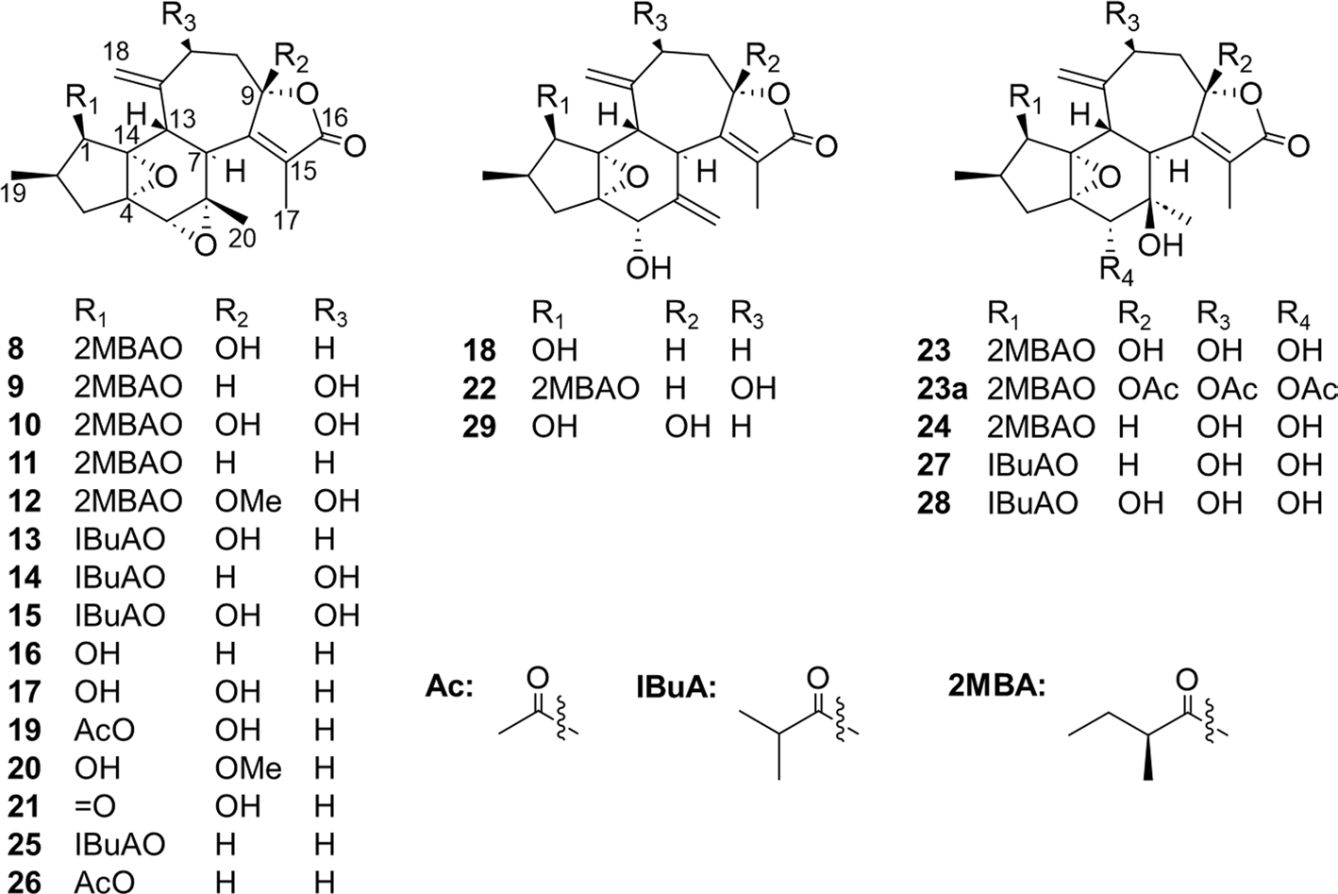
Structures of crotocascarins A‒V (**8**‒**29**)

**Fig. 3:**
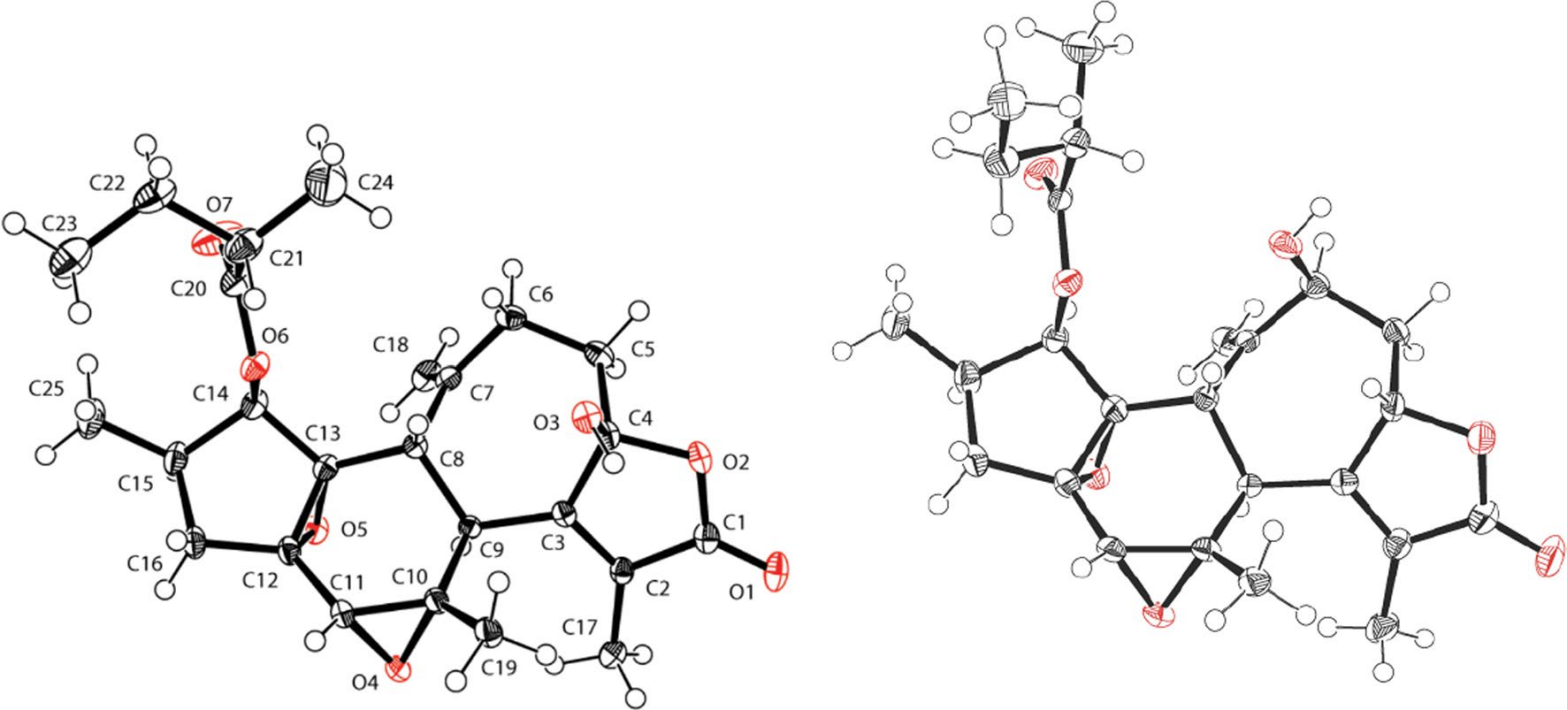
ORTEP drawings of crotocascarin A (**8**: Left) and Crotocascarin B (**9**: Right)

**Fig. 4:**
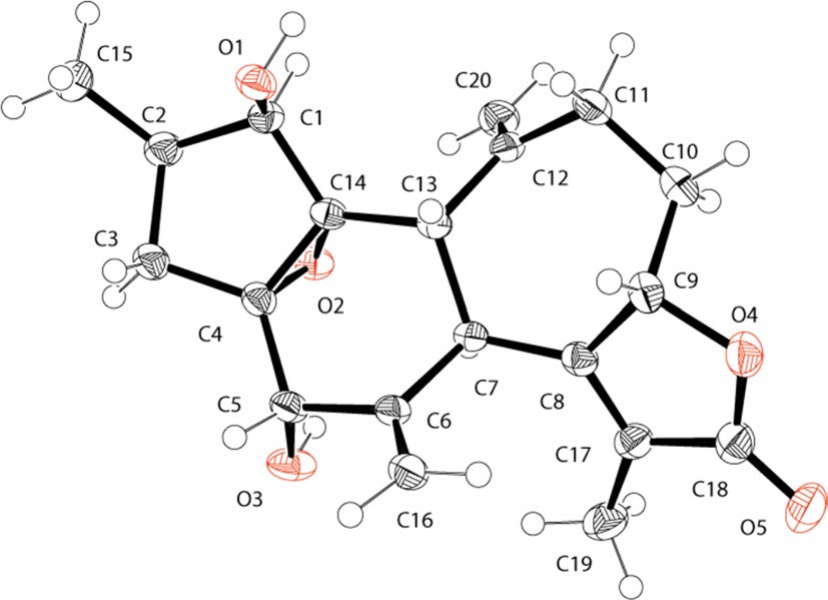
An ORTEP drawing of crotocascarin K (**18**)

**Fig. 5:**
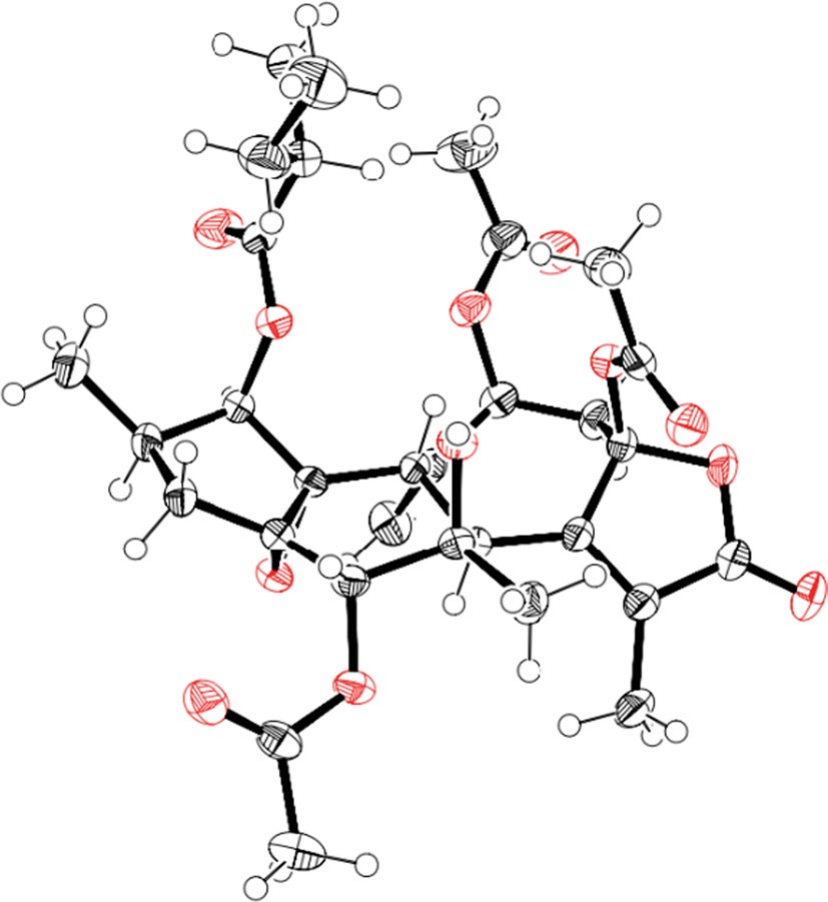
An ORTEP drawing of crotocascarin O triacetate (**23a**)

Crotofolane, which is a diterpene biosynthesized from cembrane via casbane and lathyrane through cross-annular cyclization, was first isolated as crotofolin A (**1**) in 1975 by Chan et al. from *C*. *corylifolius* [2]. After that, crotofolins B, C, and E (**2**–**4**) were isolated from *C. corylifolius* by Burke et al. in 1979 [3], crotoxides A and B (**5**, **6**) from *C*. *dichogamus* by Jogia et al. in 1989 [4], and crotohaumanoxide (**7**) from *C*. *haumanianus* by Tchissambou et al. in 1990 [5]. Only these seven crotofolanes were reported, making them difficult to be recognized as a family of compounds (Fig. 1). The structure is characterized by tricyclicity, with a fused structure of five-, six-, and seven-membered rings as the basic skeleton. As compounds of similar structure, phorbol derivatives classified in tigliane, which comprises tetracyclic diterpenes having a basic structure of fused five-, seven-, six-, and three-membered rings, are known to possess remarkable biological activity. The authors have focused on the rare diterpene crotofolanes and succeeded in isolating and determining the structures of 22 crotofolanes, as shown in Fig. 2 [6–10]. First, the structures of **8** and **9** were analyzed by the one- and two-dimensional NMR spectra, and they were deduced as crotofolanes, which comprised fused five-, six-, and seven-membered rings and an additional *γ*-lactone ring. Their relative steric structures were finally confirmed by the X-ray crystallographic technique since both compounds gave high-quality crystals for analyses (Fig. 3). Meanwhile, 2-methylbutyric acid obtained from the hydrolysis of **8** and **9** as well as the commercially available (*S*)-( +)-2-methylbutyric acid were subjected to HPLC analysis with an optical rotation detector. As a result, the absolute configuration of 2-methylbutyric acid from **8** and **9** was determined to be *S*. This experiment was the first determination attempt of the absolute configuration of crotofolane diterpenoid. In addition, for determining the absolute configuration of other crotofolanes, a more general method is used. The positive Cotton effect in the ECD spectra derived from the *α*,*β*-unsaturated *γ*-lactone moiety empirically indicated that the absolute configuration at the 9-position was *S* [11, 12]. The structures of crotofolanes with double bonds at the various positions and epoxy rings, such as **18**, **22**, **29** and **23**, **24**, **27**, **28**, respectively, were also carefully determined. Investigation of the absolute structure of compound **18** was determined by X-ray crystallographic analysis (Fig. 4), followed by application of the modified Mosher’s method [13]. Compounds **22**, **23**, **27**, and **28** were expected to be formed by the cleavage of the epoxy ring between C-5 and C-6. To confirm the geometry of these hydroxy groups; thus formed, an X-ray crystallographic analysis of crotocascarin N 5,9,11-tri-*O*-acetate (**23a**) was performed (Fig. 5), and the absolute configuration of **23** was determined to be the same as those of other crotofolanes, deduced from the positive Cotton effect at 254 nm in the ECD spectra and chirality analyses of the 2-methylbutyric acid moiety by HPLC.

## Rearranged crotofolane (nor-crotofolane, trinor-crotofolane, neocrotofolane) and novel skeletal diterpene (isocrotofolane)

**Fig. 6:**
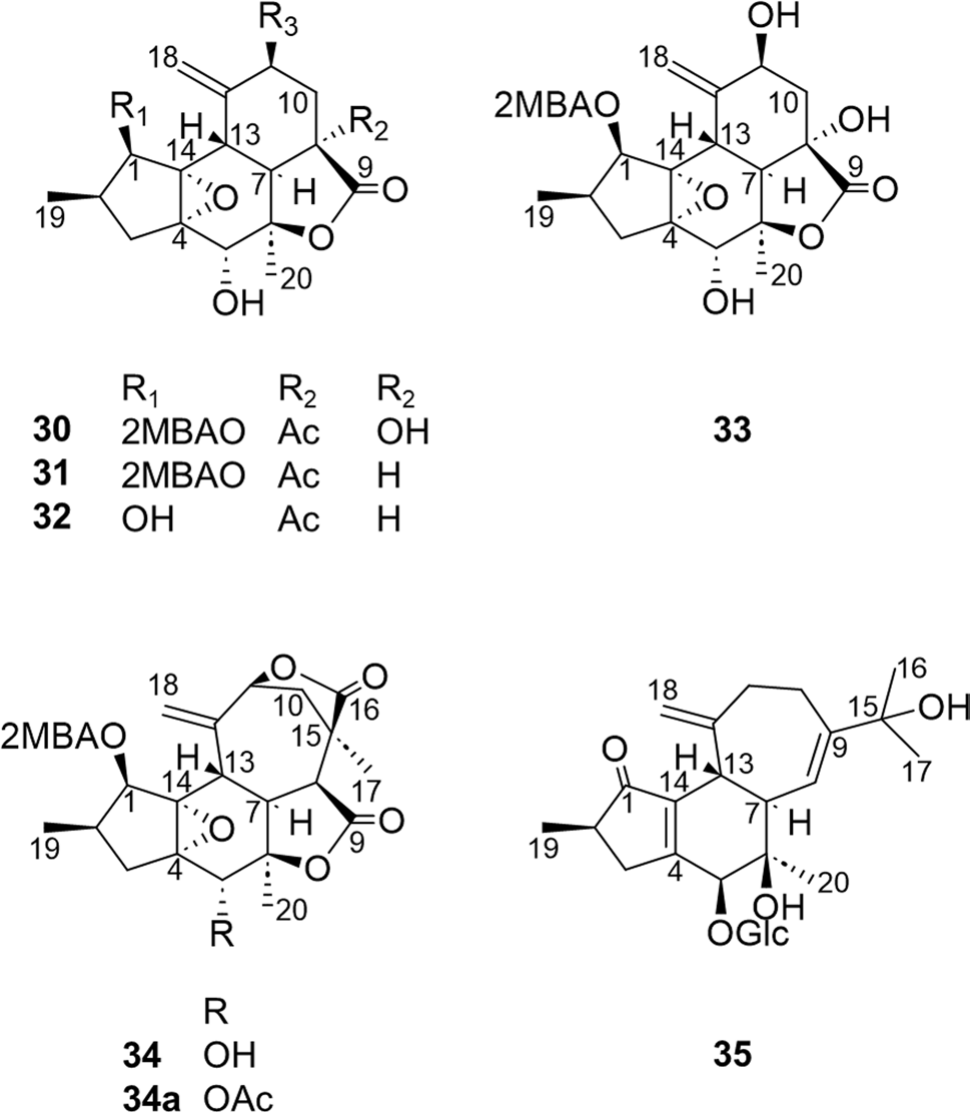
Structures of crotocascarins *α*‒*δ* (**30**‒**33**), neocrotocascarin (**34**) and isocrotoforane glucoside (**35**)

**Fig. 7:**
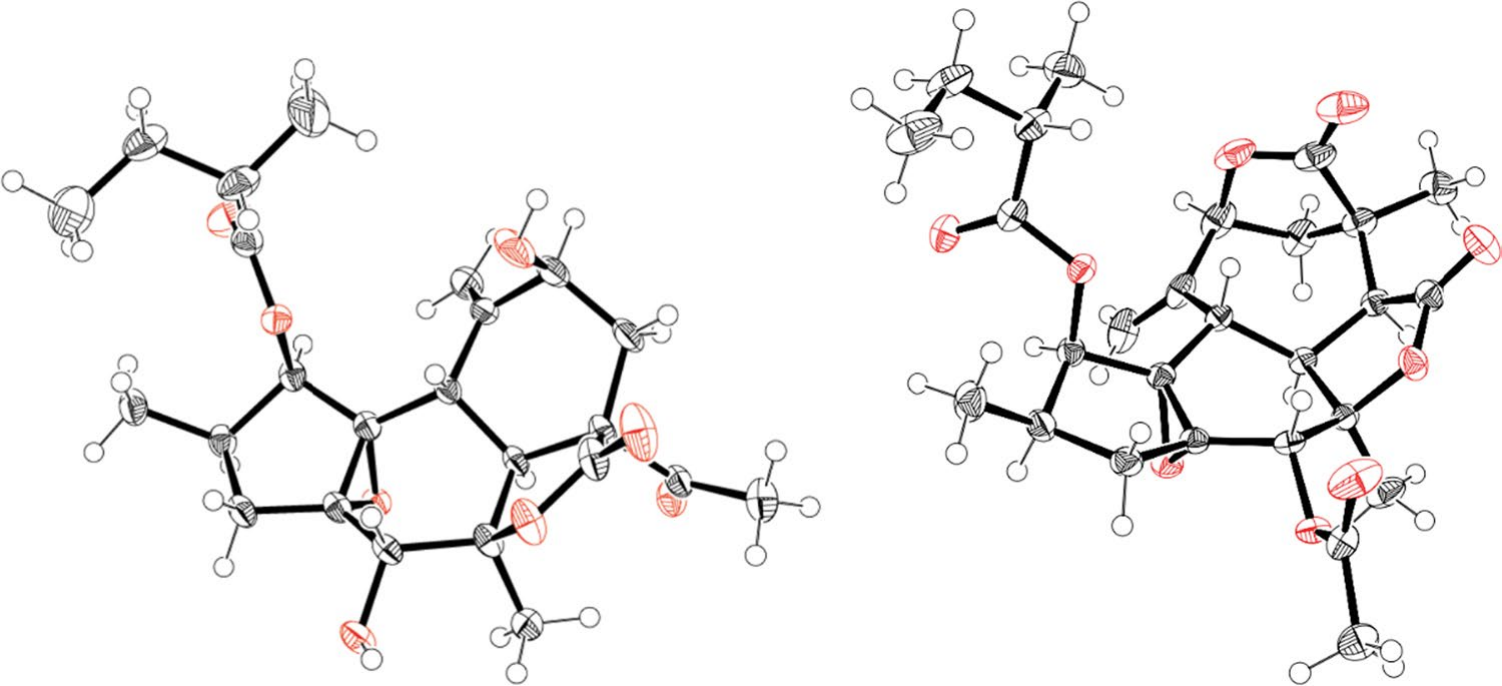
ORTEP drawings of crotocascarin *α* (**30**: Left) and neocrotocascarin acetate (**34a**: right)

**Fig. 8:**
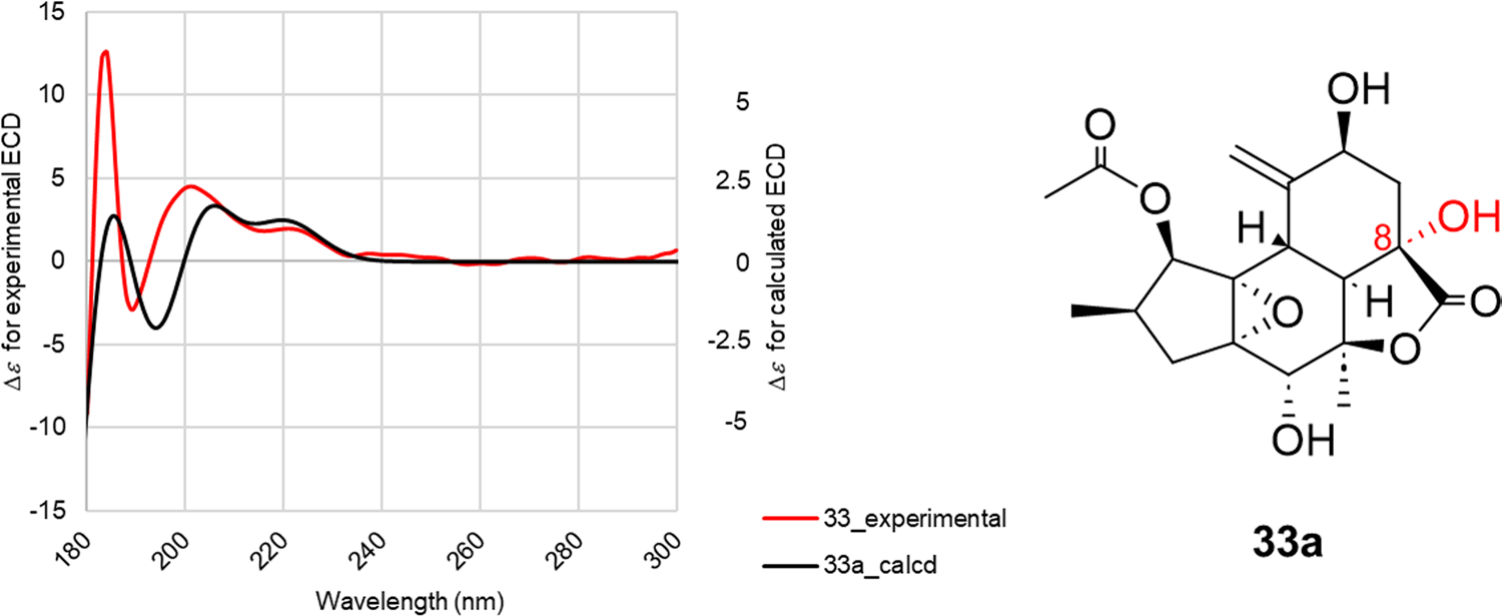
Experimental ECD spectrum of crotocascarin *δ* (**33**) (solid line in red) and calculated ECD spectra of virtual compound **33a** (solid lines in black)

The authors also report the rearranged crotofolanes nor-crotofolane [crotocascarins *α*‒*γ* (**30**–**32**)], trinor-crotofolane [crotocascarins *δ* (**33**)], and neocrotofolane [neocrotocascarin (**34**)] as well as a novel skeletal diterpenoid glycoside [isocrotofolane glucoside (**35**)], as shown in Fig. 6. The ring structures were analyzed carefully since these NMR spectra were similar to those of crotofolanes. Both **30** and **34** acetate (**34a)** were obtained as high-quality crystals, and their structures were confirmed by X-ray crystallography (Fig. 7). Thus, the structures of **30** and **34** were determined to be nor-crotofolane and rearranged crotofolane (consisting of five-, six-, and six-membered rings and a *γ*-actone fused together) and to be neocrotofolane (a rearranged crotofolane consisting of five-, six-, and seven-membered rings and two* γ*-lactones). Based on the extended NMR spectral analysis, **35** was determined to be a glycoside of isocrotofolane, a new skeleton with an isopropyl group at the 9-position, derived by a different cleavage scheme from that of the crotofolane skeleton (as described in a later section, **Consideration of biosynthetic pathways**). For **33**, in the latest report, its absolute configuration was determined by comparing the experimental ECD spectrum with the TD-DFT-calculated ECD spectrum (Fig. 8) [14].

## Consideration of biosynthetic pathways from crotofolane to nor-crotofolane, trinor-crotofolane, and neocrotofolane

**Fig. 9:**
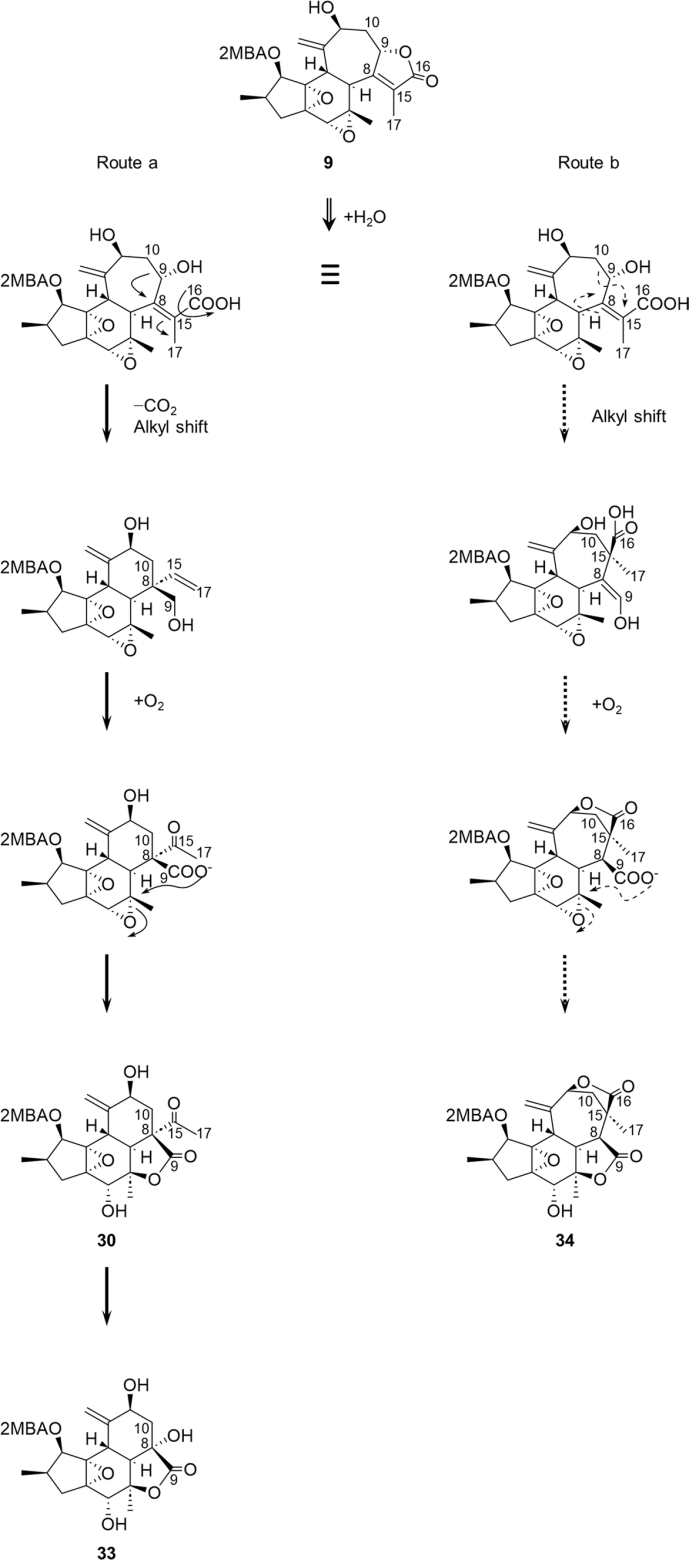
Plausible biosynthetic pathways for the formation of nor-crotofolane, trinor-crotofolane (route a: solid arrows), and neocrotocascarin (route b: dashed arrows)

**Fig. 10:**
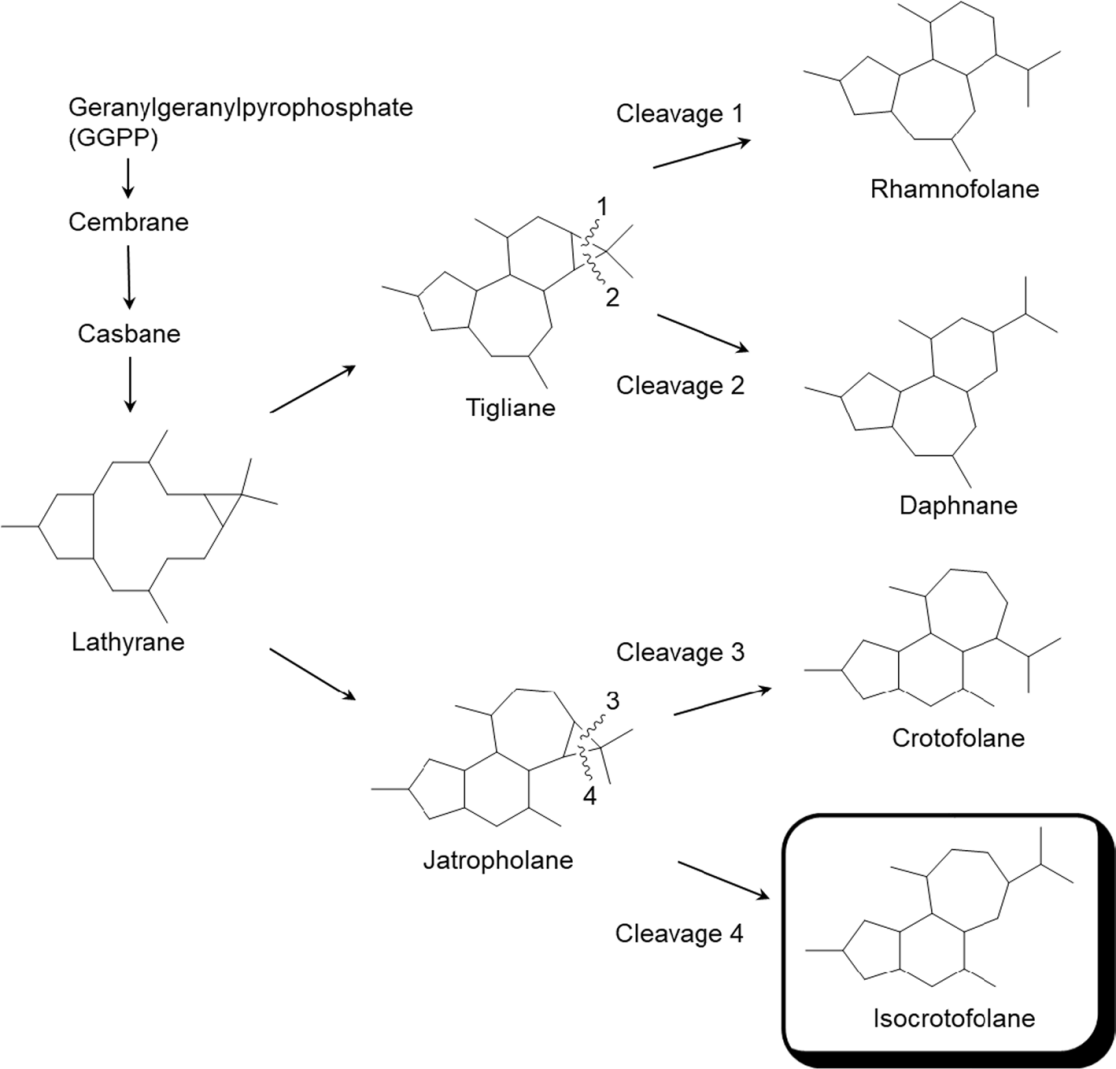
A plausible biosynthetic pathway for the formation of isocrotofolane from GGPP

The presumed biosynthetic pathways of the rearranged crotofolanes are shown in Fig. 9. Note that the biosynthetic pathways of **30** and **34** are presumed to be followed by different cyclization sites, as shown in Routes a and b (Fig. 9) during recyclization, after cleavage of the 9/10 C–C bond of **9**. Furthermore, **33** is presumed to have followed a biosynthetic pathway in which the acetyl group at the 8-position of **30** is replaced by a hydroxy group. The presumed biosynthetic pathway for the isocrotofolane skeleton is shown in Fig. 10. The relationship between rhamnofolane and daphnane derived from tigliane and the relationship between crotofolane and isocrotofolane derived from jatropholane are noteworthy in terms of biosynthetic cyclization and cleavage.

## Advancing the research on crotofolane-related compounds and their biological activities

**Fig. 11:**
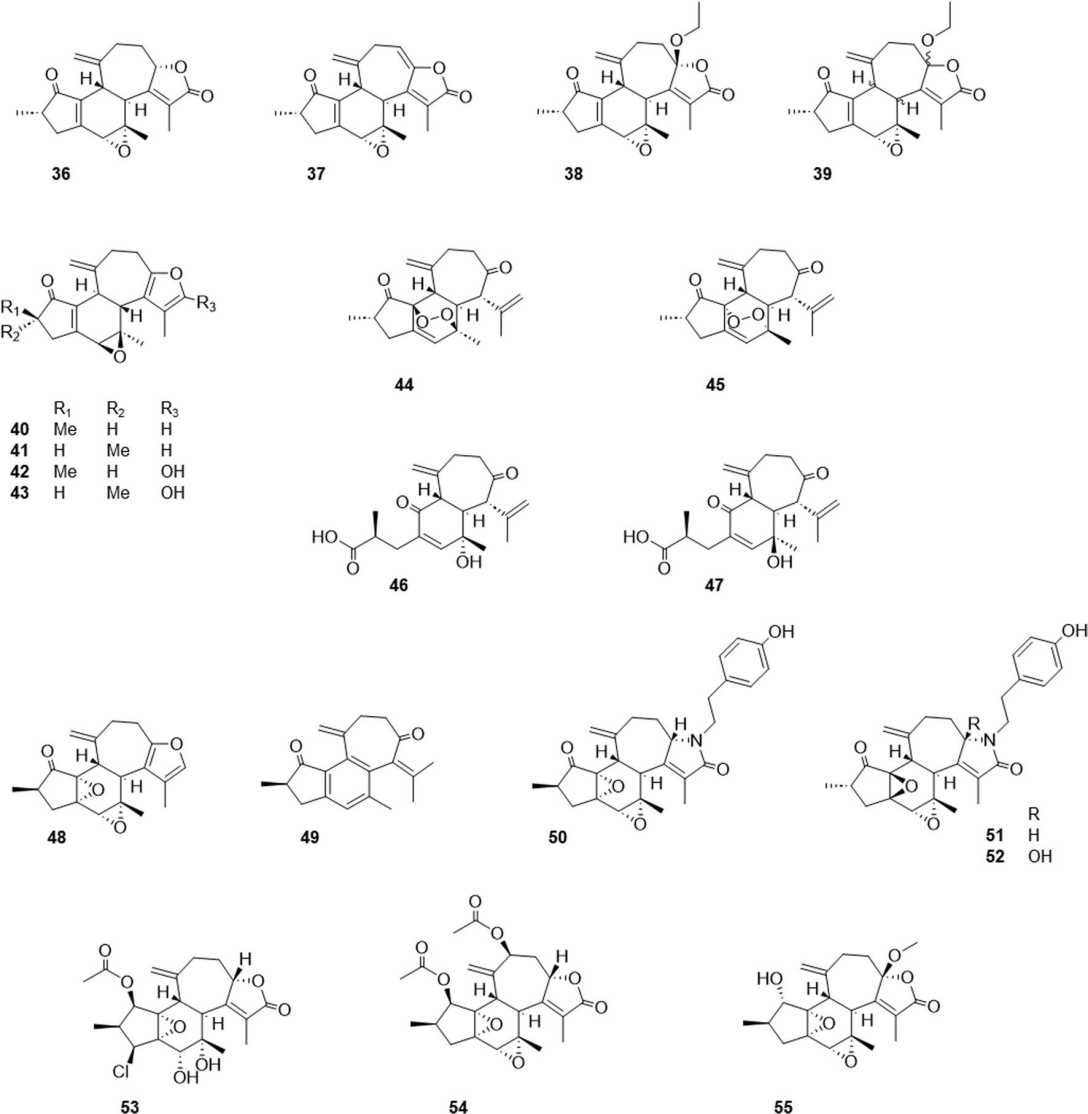
Recently reported structures of crotofolanes: crotocarasins A‒D (**36**‒**39**); Four crotofolanes (no trivial names) (**40**‒**43**); EBC-233, EBC-300, EBC-240, and EBC-241 (**44**‒**47**); crotodichogamoins A and B (**48**, **49**); cascarinoids A‒C (**50**‒**52**); 1*β*-acetoxy-3*β*-chloro-5*α*,6*α*-dihydroxycrotocascarin L and 11*β*-acetoxycrotocascarin L (**53**, **54**); and crotocascarin *ω* (**55**)

In this paper, the authors introduce new compounds with unusual structures obtained from chemical constituents research on plants native to the Ryukyu Archipelago. Only seven crotofolanes were reported before our study, although after then, other research groups began to report the isolation of crotofolanes. In 2013, Chavez et al. reported crotocarasins A–D (**36**–**39**) from *C. caracasanus* [15], and in the same year, Silva-Filho et al. reported four crotofolanes (**40**–**43**) from *C. argyrophyllus* [16]. In 2014, Maslovskaya et al. reported EBC-233, EBC-300, EBC-240, and EBC-241 (**44**–**47**), from *C. insularis* [17], while in 2017, Aldhaher et al. reported crotodichogamoins A and B (**48**, **49**) from *C. dichogamus* [18]. In 2018, Gao et al. reported cascarinoids A–C (**50**–**52**) from *C. cascarilloides* in the Yunnan Province of China [19], and in 2022, Terefe et al. reported 1*β*-acetoxy-3*β*-chloro-5*α*, 6*α*-dihydroxycrotocascarin L and 11*β*-acetoxycrotocascarin L (**53**–**54**) from *C. megalocarpus* [20]; crotocascarin *ω* (**55**) from *C. dichogamus* [21] was also reported (Fig. 11). Among them, EBC-233, EBC-300, EBC-240, EBC-241, cascarinoids A–C, and 1*β*-acetoxy-3*β*-chloro-5*α*, 6*α*-dihydroxycrotocascarin L have unique structural properties, as follows. Compounds **44** and **45** have peroxo-bridged structures between 6/14 carbons, **46** and **47** have structures with cleavage between the 1/14 carbons, **50**–**52** have structures with tyramine moiety, and **53** has a chlorine atom as a heteroatom. A recent report states that biological activities have received increasing interest in research. Compounds **51**‒**52** have been reported to exhibit immunosuppressive activity against B and T lymphocyte cell proliferation in vitro. Compounds **51** and **52** showed the inhibition of LPS-induced B-cell proliferation of IC_50_ at concentrations of 16.27 μM and 10.29 μM, respectively, and compound **52** the inhibition of ConA-induced T-cell proliferation of IC_50_ at a concentration of 6.14 μM [19]. Compounds **17**, **53**, and **55** have shown significant antiretroviral activity against HIV-1EIIIB of IC_50_ at concentrations of 28 nM, 5.5 nM, and 5.3 nM, respectively [20, 21], and the further results of these research groups are expected in the future.

## Conclusion

It has taken about half a century to elucidate the compound family of crotofolane and even some of its biological activities. The further isolation of crotofolanes will be reported in the future, and more useful biological activities will be revealed. Furthermore, because crotofolane tyramine amides [19] have also been isolated from *C*. *cascarilloides* collected in Yunnan Province, China, studies on the genetic relationship of these *C*. *cascarilloides* species are of great interest. Nor-crotofolane, trinor-crotofolane, neocrotofolane, and isocrotofolane, which are candidates for new groups of natural compounds discovered by the authors, as has crotofolane, are expected to be increasingly isolated, to be recognized as new groups of natural compounds, and furthermore, to possess important biological activities.
